# Estimating the accuracy of calculated electron paramagnetic resonance hyperfine couplings for a lytic polysaccharide monooxygenase

**DOI:** 10.1016/j.csbj.2020.12.014

**Published:** 2020-12-20

**Authors:** Yusuf A. Theibich, Stephan P.A. Sauer, Leila Lo Leggio, Erik D. Hedegård

**Affiliations:** a Department of Chemistry, University of University, Copenhagen, Denmark; b Division of Theoretical Chemistry, Lund University, Chemical Centre, P. O. Box 124, SE-221 00 Lund, Sweden

**Keywords:** LPMO, QM/MM, EPR, DFT

## Abstract

Lytic polysaccharide monooxygenases (LPMOs) are enzymes that bind polysaccharides followed by an (oxidative) disruption of the polysaccharide surface, thereby boosting depolymerization. The binding process between the LPMO catalytic domain and polysaccharide is key to the mechanism and establishing structure-function relationships for this binding is therefore crucial. The hyperfine coupling constants (HFCs) from EPR spectroscopy have proven useful for this purpose. Unfortunately, EPR does not provide direct structural data and therefore the experimental EPR parameters have to be supported with parameters calculated with density functional theory. Yet, calculated HFCs are extremely sensitive to the employed computational setup. Using the LPMO *Ls*(AA9)A catalytic domain, we here quantify the importance of several choices in the computational setup, ranging from the use of specialized basis, the underlying structures, and the employed exchange-correlation functional. We show that specialized basis sets are an absolute necessity, and also that care has to be taken in the optimization of the underlying structure: only by allowing large parts of the protein around the active site to structurally relax could we obtain results that uniformly reproduced experimental trends. We compare our results to previously published X-ray structures and experimental HFCs for *Ls*(AA9)A as well as to recent experimental/theoretical results for another (AA10) family of LPMOs.

## Graphical abstract

**Figure:**
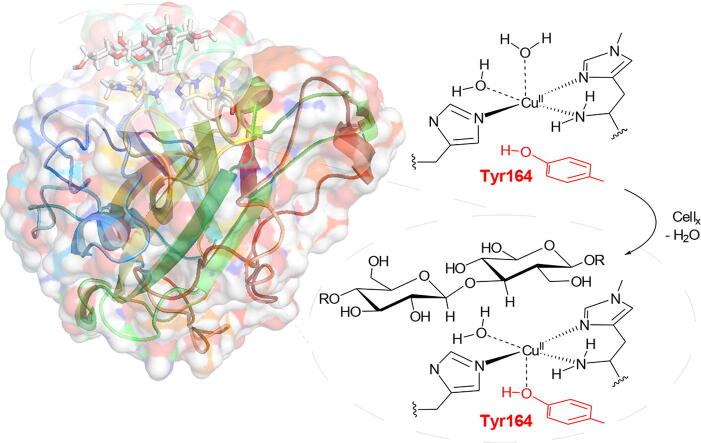

## 1 Introduction

The discovery of new copper enzymes boosting depolymerization of polysaccharides [1], [2], has further fueled the hope of exploiting the vast carbon resource of naturally occurring (but mostly recalcitrant) polysaccharides [3]. An obvious utilization is biofuel production but also commercial chemicals would be a rewarding target. The enzymes responsible for the boost belong to families of auxiliary activity [4] (AA) enzymes and are denoted lytic polysaccharide monoxoygenases (LPMOs) [1], [2]. Their auxilliary activity is associated with oxidation of the glycoside link in polysaccharides [2] leading to disruption of the (crystalline) polysaccharide surface with concomitant boost in polysaccharide decomposition [5].

**Fig. 1:**
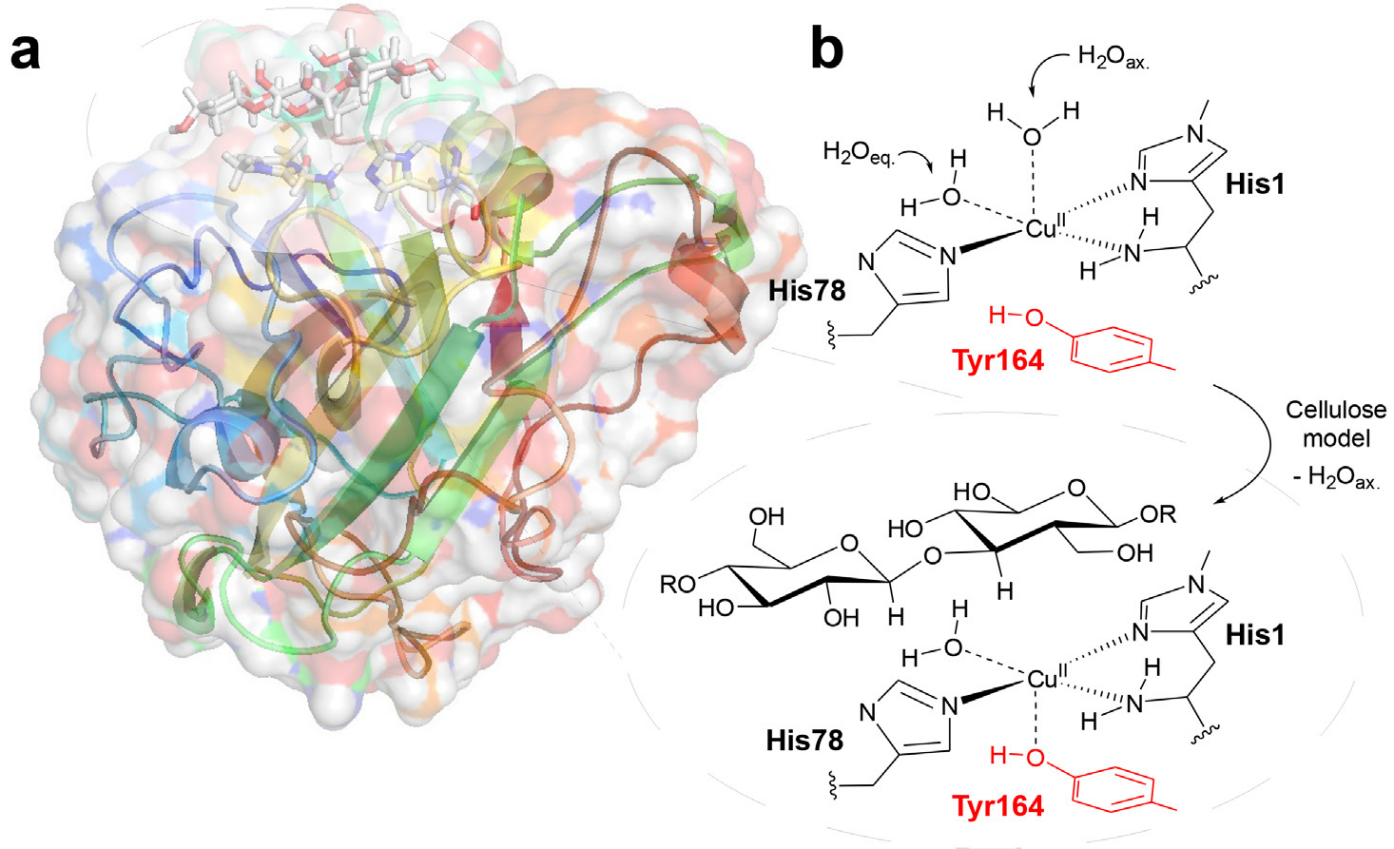
(a) Structure of Ls(AA9)A (PDB: 5ACF) with substrate bound [6]. (b) The histidine brace without substrate and the postulated changes occurring when a substrate binds (displacement of water and tighter binding of Tyr164).

A number of different LPMO catalytic domains have been categorized, belonging to the distinct families, AA9-AA16 (with AA12 exempted) [2], [1], [7], [8], [9], [10], [11], [12], [13]. The overall structures of the LPMOs are similar, although amino-acid sequences for the different LPMO families vary considerably; they also target a wide range of different polysaccharide substrates with different regio- and stereo-specificities [14], [15], [16], [17]. The most important common features are the overall fold, a large, flat substrate-binding surface, and an active site with a copper ion [7], coordinated by two histidine residues (see Fig. 1). This motif has become known as the histidine brace, in which one histidine is the amino-terminal residue that coordinates bidentate through the N-terminus and the imidazole side chain.

The mechanism behind LPMOs’ remarkable reactivity is still heavily debated. For instance, the nature of the species responsible for oxidation of the glycoside link is not clarified [18], [19], [20], [21], [22], [23], [24], [25], [26], [27], [28]. The co-substrate is also debated as both O_2_ and H_2_O_2_ have been suggested as the natural co-substrate [29], [30]. Another crucial part of the mechanism is the initial substrate binding on the LPMO surface. The LPMOs often target insoluble polysaccharide substrates, making study of the binding a large challenge, requiring both different techniques as well as theoretical models. On the experimental side, site-directed mutagenesis [31], [1], [32] has been employed to single out important amino acids on the binding surface. The binding process has also been studied directly by nuclear magnetic resonance (NMR) [33], [34] although it is often necessary to resort to Zn-loaded or apo-proteins since copper can obscure the spectra.

Complementary information to NMR can be obtained with electron paramagnetic resonance (EPR) in the Cu(II) state: The different LPMO families usually exhibit EPR spectra characteristic of so-called type 2 copper sites [35]. In type 2 copper sites, the (axially) distorted structures give g-values over 2, a pattern with $g_{z}>g_{x}\approx g_{y}$, and hyperfine coupling tensors with large A_z_ values ($A_{z}>A_{x}\approx A_{y}$). The axial EPR spectra are a direct consequence of the Jahn-Teller distorted resting state with Cu(II) bound to (in addition to the histidine brace) two water molecules, placed in equatorial and axial positions to a tyrosine OH group (H_2_O_eq._, H_2_O_ax._ and Tyr164 in Fig. 1b). An exception is the EPR spectra of AA10 LPMOs: while they may still be considered axial, the AA10 LPMOs usually have the active site tyrosine replaced by phenylalanine, and hence AA10 EPR spectra display substantial broadening and more rhombicity (i.e. different g- and A-tensor values) than other LPMOs [36]. The other LPMO families have active site tyrosines, but EPR data for AA11-AA15 LPMOs are more sparse and occasionally ambiguous [12], [37], [38]. Still, they can generally also be considered as type 2 copper sites.

Employing EPR, geometrical- and electronic-structure changes have been linked to the substrate binding process [39], [40], [41], [42], through spectral perturbations on substrate addition: Borisova et al. [39] obtained increased g_z_- and $\left|A_{z}\right|$-values after adding substrate to an AA9 LPMO. Similar changes have been observed in other studies [40], [43] and have been associated with displacement of the axial water molecule (H_2_O_ax._ in Fig. 1b) and tighter binding of the axial tyrosine [40]. Perturbation of EPR spectra upon substrate addition was also recently reported for the AA10 *Sm*AA10A [41]. Although not all LPMOs display these pertubations [7], [11], [36], the observed changes of the EPR spectra have been interpreted to indicate a change in electronic structure that prepare the active site for interaction with the co-substrate (O_2_ or H_2_O_2_). [40] This point has further been elaborated in an integrated NMR and EPR study, suggesting that substrate binding and O_2_-activation mechanisms are coupled [44].

In several of the recent investigations [41], [44], experimental EPR spectra were complemented by spin-Hamiltonian parameters calculated by density functional theory (DFT), thus providing an interesting new angle to the study of LPMO substrate binding. Theoretical studies of the binding process have otherwise mainly been carried out using (classical) molecular dynamics (MD) or docking [45], [46], [39], [34]. Calculation of EPR parameters have previously been applied to blue copper proteins [47], [48] and can perhaps also provide new evidence for changes associated to substrate binding for LPMOs. Still, it is well known (as also stressed in Refs. [41], [44]) that calculated EPR parameters are extremely sensitive to the computational setup [49], [50], [51], [52], [53], [54], [55], [56], [57], [58]. Here we will therefore quantify the sensitivity of calculated EPR parameters to typical choices made in the computational setup: we investigate the role of the underlying structure, i.e., the size of the model used to represent the active site and whether relaxing a larger part of the surrounding protein during structure optimization has an effect. We additionally investigate to what extend specialized basis sets are required and how large the effect of the employed DFT functional is. Our focus is here on Cu(II) hyperfine couplings (HFCs) and ligand (super) HFCs. We target the AA9 *Ls*(AA9)A since for this system we can compare both calculated QM/MM structures, and EPR parameters with experimental counterparts [40]. Finally, our study can also indicate if the recent results from AA10 [41], [44] are transferable to AA9 LPMOs.

## 2 Computational details

### 2.1 QM/MM structure optimizations

**Fig. 2:**
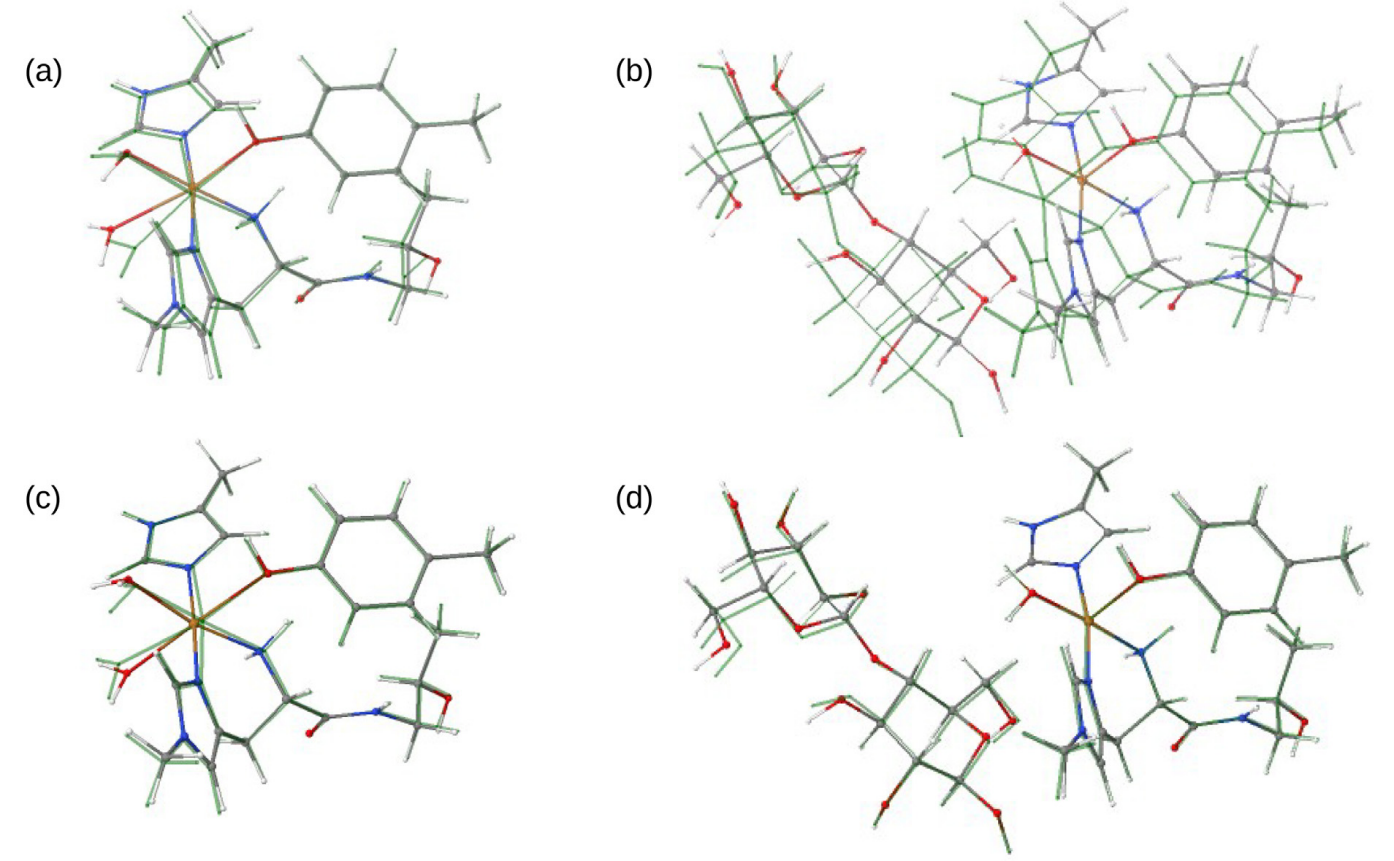
QM regions for optimizations with fixed and free MM regions, respectively. Figures (a) and (b) show overlay of QM/MM TPSS-D3/def2-SV(P) optimizations with the MM region fixed (green) and free (colored) for optimizations without (a) and with (b) substrate. Figures (c) and (d) show overlay of QM/MM optimized structures with free MM region for TPSS-D3/def2-SV(P) (green) and TPSS-D3/def2-TZVPD (colored) for optimizations without (c) and with (d) substrate. For interpretation of the references to colour in this figure legend, the reader is referred to the web version of this article.

The employed optimized structures for the active site were constructed from QM/MM optimizations based on the LPMO-substrate complex crystal structure of *Ls*(AA9)A [40] (PDB 5ACF). For both the substrate-bound complex and the LPMO without substrate, we have in most cases employed optimized structures obtained previously [27], [59] and we refer to these studies for more explicit details regarding the optimizations (a few structures were optimized for this work, but the setup was identical to the ones presented in Refs. [27], [59]). All structure optimizations were performed with an electrostatic embedded QM/MM approach, using the QM software Turbomole 7.1 [60] and the MM software AMBER 14 [61]. The QM/MM calculations were performed with the ComQum interface [62], [63], which combines these two programs. The QM/MM optimizations employed DFT as QM method in form of dispersion corrected TPSS-D3 [64], [65] with Becke-Johnson damping [66] together with a def2-SV(P) basis set [67], [68]. Additional optimizations were also carried out with the def2-TZVPD basis set. The protein was described with the Amber FF14SB force field [69] and water molecules with the TIP3P model [70]. When the substrate was included in the MM part, it was described by the glycam.v06 force field [71]. The optimizations were carried out with both MM region frozen and with residues within 6 Å of the QM region structurally relaxed (at the MM level). The QM system is comprised of Cu, the first coordination sphere and parts of the substrate (see Fig. 2). In the following, we used the labels “fixed” and “free” for calculations with the MM system fixed or partly relaxed, respectively. Note that all QM/MM optimizations were performed without second-sphere His147 and Gln162 residues in the QM region (which were included in most of our earlier studies [27], [59], as they were involved directly in the mechanisms investigated).

For the optimizations without substrate, the substrate was removed and the protein re-equilibrated (with identical procedure as described in Refs. [72], [27], [59]), allowing a water molecule to bind to the solvent-exposed active site. The structures were then QM/MM optimized with an active site identical (apart from the substrate) to the optimizations with substrate. A slight difference from Ref. [59] (apart from that the residues Gln162 and His147 were not included) is that both “fixed” and “free” optimizations were performed for this work.

### 2.2 EPR parameter calculations

**Fig. 3:**
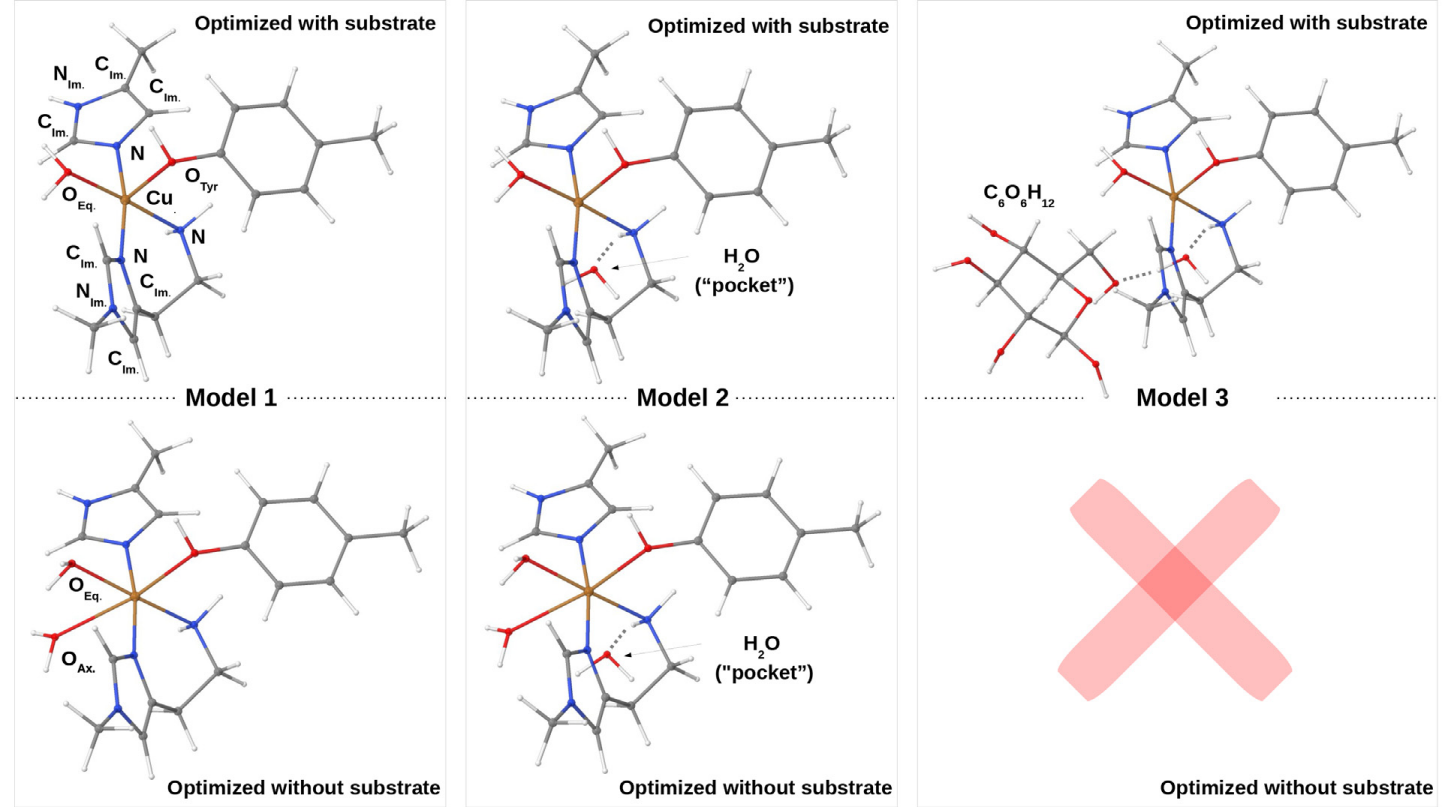
Different model sizes in the study of EPR parameters; all are based on QM/MM optimizations with slightly larger QM regions (see Fig. 2). The shown structures are from optimizations with the MM regions relaxed. For model 1 an additional set of structures were obtained from QM/MM optimizations in Fig. 2 with MM region unrelaxed. The differences between the models are highlighted with labels (model 1 optimized with substrate also shows labels used for the basis set study). The pocket water molecule introduced from model 2 and onward was not part of the QM region and was therefore not shown in Fig. 2.

From fixed and free QM/MM optimizations, respectively, we cut out three different sizes of systems (models 1–3) on which calculations of EPR parameters were carried out. The different models are shown in Fig. 3. We calculated both HFCs of Cu and coordinating N atoms (note that the MM charges are not included in the EPR parameter calculations and hence only structural effects of the protein are accounted for). For Cu, the isotropic Fermi contact term, the anisotropic spin-dipolar contribution, as well as the spin-orbit coupling contributions were calculated. The contributions from spin-orbit coupling are often non-negligible for Cu [50], [53], [73], [74], but increase the computational effort considerably: they are calculated as a linear response function of the paramagnetic spin-orbit and the spin-orbit coupling operators. This is also why small models are required for the larger basis sets. Spin-orbit coupling can safely be neglected for the super HFCs of the nitrogen atoms and here only the two first-order terms were evaluated.

The calculations of the EPR hyperfine and super HFCs were carried out with the ORCA program version 4.1.1 [50], [52], [75], [76]. Before investigating model 1–3 with a range of functionals, we investigated the use of specialized core-property basis sets. For this basis set study, we grouped the atoms in five groups, in short Cu/{N&O_Eq._& O_Tyr_}/{C_Im_&N_Im_}/C/H (see Fig. 3). Thus, the Cu atom is the first group. In the second group the nitrogen and oxygen atoms coordinating the Cu atom were included. The third group consists of the carbon and other nitrogen atoms in the imidazole rings (labelled “Im.” in Fig. 3), while the fourth group includes all other carbon atoms. All hydrogen atoms, finally, constitute the fifth group. We then employed the specialized core-property basis set aug-cc-pVTZ-J [77], [78], [79], [80], [81], [82] either on all atoms or only on some atoms in a locally-dense basis set fashion [83], [84], [85], [58], [86] together with the standard correlation consistent basis sets cc-pVDZ, cc-pVTZ and aug-cc-pVTZ [87], [88], [89] on the other atoms. Denoting the basis sets aug-cc-pVTZ-J, aug-cc-pVTZ, cc-pVTZ and cc-pVDZ as aTJ, aT, T and D, we obtain a series of six basis sets, systematically decreasing in size: B6 (aTJ/aTJ/aTJ/aTJ/aTJ), B5 (aTJ/aTJ/aTJ/aTJ/D), B4 (aTJ/aTJ/aTJ/aTJ/D), B3 (aTJ/aTJ/aTJ/T/D), B2 (aTJ/aTJ/T/T/D), B1 (aTJ/T/T/T/D).

Additional calculations with the completely decontracted def2-TZVP and def2-TZVPP basis sets [90] were carried out as these basis sets have been employed previously for LPMOs [41]. A set of calculations was also performed exclusively with standard correlation consistent basis sets but these will not be discussed in detail (see Tables S2 and S3 in the SI).

We selected B3 to carry out calculations on model 1–3 (based on free QM/MM optimizations) with the GGA functionals BLYP [91], [92] and PBE [93], the hybrid GGA functionals B3LYP [94] and PBE0 [95], [96], [97], the meta-GGA functional TPSS [64] and the hybrid meta-GGA functional TPSSh [64] (note that the basis set investigation was carried out using only the B3LYP functional, employing model 1, based on QM/MM optimization with relaxed MM region). In addition, a set of calculations (with all functionals) were done for model 1, based on QM/MM optimizations with unrelaxed MM region. In all calculations of HFCs, a large integration grid for the exchange-correlation functional (Grid7) was employed.

## 3 Results and discussion

Calculation of EPR parameters are known to be sensitive to the computational setup, including the underlying structures. The first section therefore discusses the relevant structures, which all are obtained with QM/MM, but employing different QM/MM optimization protocols (as described under Computational Details). In two subsequent subsections, we discuss the basis set, the effect of the chosen DFT functional, and the effect of employing models sizes beyond the first coordination sphere of the copper ion. In all cases, we also compare our calculated results against previously published experimental data, as indicated by references in the appropriate tables. Finally, we discuss our findings in relation to recent studies of the substrate binding process, as well as the compilation of experimental EPR parameters for LPMOs provided by Vu and Ngo [38].

### 3.1 Structures

We start with comparing structures obtained with TPSS-D3/def2-SV(P) and the MM region structurally relaxed (“free”) and unrelaxed (“fixed”): the optimized structures of the *Ls*(AA9)A active site with these specifications are shown in Fig. 2 both without (a) and with (b) substrate; structures obtained with fixed MM region are shown in green. Selected bond distances are given in Table 1, including results from previous QM/MM optimizations for *Ta*LPMO9A [72]. It has previously been shown [72], [27] that employing a relaxed MM region only leads to minor effects for intermediates later in the catalytic cycle (after O_2_ binds), whereas relaxing the MM region can lead to large differences for both Cu(II) resting state and the Cu(I) state obtained after initial reduction. The results in Fig. 2 and Table 1 confirm this observation for the Cu(II) resting state, where differences are particularly large for substrate-bound structures.

A closer look at the obtained distances between the copper center and the first coordination sphere for both optimizations, shows that the Cu–N bonds changed minimally, whereas the Cu–O bonds are more sensitive, particularly, bonds along the Jahn-Teller axis, i.e., $\text{Cu}-{\text{O}}_{W}^{\mathrm{ax}.}$ and Cu–O_Tyr_. The Cu–O_Tyr_ bond is generally shorter in the optimizations with parts of the MM region relaxed, but in both cases, Cu–O_Tyr_ decreased upon substrate binding: the bond changed from 2.34 Å to 2.24 Å without substrate, while the corresponding change is 2.42 Å and 2.29 Å with the MM region fixed.

When comparing with *Ls*(AA9)A crystal structures with and without substrate (PDB: 5ACF and 5ACG), it should be noted that although they represent low-dose X-ray structures with minimal photoreduction, partial reduction cannot be excluded. Moreover, the substrate-bound crystal structure (5ACF) binds Cl^-^ instead of the equatorial water, meaning that direct comparison is precluded. With these precautions in mind, we still comment on the optimized structures in comparison with the crystallographic results: qualitatively, optimizations with fixed and free MM region both led to a reduction of the Cu–O_Tyr_ bond distance upon substrate binding (0.1 Å), similar to what has been shown experimentally in crystal structures (0.2 Å) [40]. Despite this good agreement, larger differences were seen when comparing the absolute distances for the Cu–O bonds, while Cu–N distances were in quite good correspondence with the crystal structures. The calculated values for Cu–O_Tyr_ were generally shorter than the experimental values, both with and without substrate bound.

Meanwhile, the bond distance for the axial water molecule changed from 2.25 Å (MM fixed) to 2.53 Å (MM relaxed), the latter closer to the experimentally [40] obtained 2.8 Å, but still significantly off. From these results, it seems that the calculations with relaxed MM region obtained structures where both Cu–O_Tyr_ and $\text{Cu}-{\text{O}}_{W}^{\mathrm{ax}.}$ were elongated compared to the remaining coordination bonds. This has also been observed experimentally, albeit the calculated elongation was here found to be less pronounced than seen in the crystal structure [40]. We have previously noted such differences for the weak Cu–O bonds in LPMOs [72] and a selection of previous QM/MM results are collected in Table 1 (including results from another AA9 LPMO, *Ta*LPMO9A). These results show similar variations among tyrosine and axial water Cu–O bonds, with significant differences both within and between theoretical and crystallographic results. Even larger variations are found, if we also include previous QM-cluster results [20], [98] but these have already been discussed in detail in earlier publications [72]. Interestingly, results with relaxed MM regions led for both *Ls*(AA9)A and *Ta*LPMO9A, to elongation of the $\text{Cu}-{\text{O}}_{W}^{\mathrm{ax}.}$ bond and shortening of the Cu–O_Tyr_ bond.

**Table 1:** Cu–ligand bond lengths (Å) for the active site of LPMO (see also Fig. 2). For brevity, we denote TPSS-D3 and B3LYP-D3 as TPSS and B3LYP, respectively, while we denoted def2-SV(P) and def2-TZVPD as SV(P) and TZVPD.

QM//MM model	$\text{Cu}-{\text{N}}_{\mathrm{His}78}^{∊}$	Cu–N_His1_	$\text{Cu}-{\text{N}}_{\mathrm{His}1}^{\delta }$	Cu–O_Tyr_	$\text{Cu}-{\text{O}}_{W}^{\mathrm{ax}.}$	$\text{Cu}-{\text{O}}_{W}^{\mathrm{eq}.}$
	Optimized without substrate *Ls*(AA9)A					
TPSS/SV(P)//Fixed	1.99	2.11	1.97	2.42	2.25	2.16
TPSS/SV(P)//Free	2.03	2.07	2.00	2.34	2.53	2.08
TPSS/TZVPD//Free	2.00	2.09	1.97	2.61	2.34	2.21
Exp. 5ACG [40]	2.1	2.2	1.9	2.7	2.8	2.2

	Optimized with substrate *Ls*(AA9)A					
TPSS/SV(P)//Fixed [27]	1.99	2.09	1.95	2.29	–	2.17
TPSS/SV(P)//Free [27]	2.06	2.04	2.00	2.24	–	2.04
TPSS/TZVPD//Free	2.04	2.02	1.98	2.26	–	2.03
Exp. 5ACF [40]	2.1	1.9	2.2	2.5	–	–

	Optimized without substrate *Ta*LPMO9A					
TPSS/SV(P)//Fixed [72]	2.02	2.07	1.98	2.80	2.28	2.11
TPSS/SV(P)//Free [72]	2.03	2.03	1.99	2.34	2.83	2.03
TPSS/TZVPD//Free [72]	2.02	2.02	1.97	2.48	3.00	2.06
B3LYP/TZVPD//Free [72]	2.04	1.98	2.02	2.47	2.96	2.07
Exp. 2YET [7]	2.32	2.10	2.43	2.80	2.65	2.23
Exp. 3ZUD [7]	2.03	2.20	1.91	2.92	2.89	–

In an additional set of calculations, we also optimized structures with a larger basis set, including diffuse functions (def2-TZVPD), still allowing the MM region to structurally relax. The obtained structures are compared to structures overlayed with structures obtained with def2-SV(P) in Fig. 2 for substrate-bound (c) and unbound (d) states, respectively. Selected distances are also given in Table 1: the substrate-bound structure turned out to be rather unaffected by the use of a larger basis set, while larger changes were seen for the *Ls*(AA9)A structure without substrate: the Cu–O_Tyr_ distance changed from 2.34 Å to 2.61 Å, in better agreement with the experimental value of 2.7 Å. Meanwhile, the distance to $\text{Cu}-{\text{O}}_{W}^{\mathrm{ax}.}$ became shorter (from 2.53 Å to 2.34 Å) which is in worse agreement with the crystal structure (2.8 Å). The distance to the equatorial water molecule, $\text{Cu}-{\text{O}}_{W}^{\mathrm{eq}.}$ also changed from 2.08 Å to 2.21 Å, where the latter is in better agreement with the crystallographic result (2.2 Å). The differences obtained for Cu–O bonds between structures optimized with def2-SV(P) and def2-TZVPD (both with the MM region partly relaxed) are not negligible, but the latter is worse for reproducing the EPR hyperfine coupling, as will be discussed further in a section below. In addition to bond-distances, we have also compiled selected bond angles around the Cu atom in Table S1 in the SI. Experimentally, the (selected) angles are all close to 90°, and the calculated values generally comes close, with smaller variations as seen for the bond distances. The largest differences to experiment (8°) is for the structure optimized with MM region fixed (see ${\text{N}}_{\mathrm{His}78}^{∊}-\text{Cu}-{\text{O}}_{\mathrm{Eq}.}^{W}$ in Table S1).

In conclusion, the structure (particular the Cu–O distances) can depend significantly on the strategy employed in the optimization, and we will therefore investigate all structures in EPR parameter calculations to quantify how large an effect the strategy employed in QM/MM optimizations has on the obtained parameters. However, we first investigated the influence of the employed basis set which is described in next section.

### 3.2 Basis set study of hyperfine coupling parameters

The basis sets regularly employed in quantum chemical calculations are not optimized to be accurate in the atomic core region. Hence, the part of the EPR spin-Hamiltonian, depending on the core spin-density (i.e. the HFCs) can become erratic, if not specially designed core-property basis sets are employed [99], [100], [77], [78], [79], [80], [81], [82], [54], [101], [102], [103], [104], [105], [106], [107], [108], [109]. An alternative is to decontract a regular basis set to make it more flexible in the core region. Here we discuss both strategies, employing the aug-cc-pVTZ-J basis sets [77], [78], [79], [80], [81], [82], [54] as well as the decontracted def2-TZVP and def2-TZVPP basis sets [90] (which were employed in Ref. [41]). Since both strategies result in rather large basis sets, we additionally investigated a locally-dense strategy, where aug-cc-pVTZ-J was employed locally on the nuclei of interest, while smaller basis sets were employed for the remaining nuclei. We and others have previously shown this strategy to work well [83], [84], [85], [58], [86] also for systems containing transition metals [58]. However, to the best of our knowledge, no systematic studies have yet been performed to test if this strategy works well for LPMOs (or indeed any other copper proteins).

We chose to carry out the basis set study on the smallest model without substrate, i.e., model 1 in Fig. 3; optimized with TPSS-D3/def2-SV(P) and the MM region relaxed. The effect of enlarging the model on the calculated HFCs was also investigated and will be described in next subsection. We constructed several locally-dense basis sets with aug-cc-pVTZ-J on an increasing number of atoms and smaller, standard Dunning basis sets on the other atoms (B1–B6, see Computational Details).

The results from the basis set investigation are compiled in Table 2 for the copper atom and Table 3 for the nitrogen atoms in the first coordination sphere. We consider the results with the aT/aT/aT/aT/aT combination of basis sets as best values obtained with standard basis sets and results with B6 are the overall best values obtained.

**Table 2:** Effect of employed basis on the three principal components (A_11_–A_33_)${}^{a)}$ of the Cu HFC (in MHz) with B3LYP (model 1 in Fig. 3, optimized without substrate and TPSS-D3/def2-SV(P) with the MM region relaxed). The contributions from the Fermi contact (A_FC_) and spin–orbit ($A_{\mathrm{iso}}^{\mathrm{SO}}$) terms are also given. CGTOs is the number of contracted Gaussian functions in the basis set.

Basis	# CGTOs	A_FC_	$A_{\mathrm{iso}}^{\mathrm{SO}}$	A_11_	A_22_	A_33_	A_iso_
							
aT/aT/aT/aT/aT	1910	198.2	138.3	−89.6	509.2	589.7	336.5

B1: aTJ/T/T/T/D	1016	−252.6	135.7	47.4	126.7	−524.8	−116.9
B2: aTJ/aTJ/T/T/D	1112	−251.9	135.8	47.9	127.7	−524.0	−116.1
B3: aTJ/aTJ/aTJ/T/D	1240	−252.2	135.8	47.7	127.4	−524.3	−116.4
B4: aTJ/aTJ/aTJ/aTJ/D	1416	−257.1	135.7	42.7	122.4	−529.3	−121.4
B5: aTJ/aTJ/aTJ/aTJ/T	1677	−252.3	135.8	47.8	127.1	−524.5	−116.5
B6: aTJ/aTJ/aTJ/aTJ/aTJ	1851	−252.3	135.8	47.8	127.1	−524.5	−116.5

TZVP-uncontracted	1474	−245.4	133.3	51.8	124.0	−512.1	−112.1
TZVPP-uncontracted	1725	−245.2	132.4	53.4	127.5	−512.2	−112.8

Exp. [40]	–	–	–	58	78	−458	−107

${}^{a)}$ A_11_ is aligned with the N_His1_$-\text{Cu}-{\text{O}}_{W}^{\mathrm{eq}.}$ bonds; A_22_ is aligned with the $N_{\mathrm{His}78}^{∊}$–Cu–${\text{N}}_{\mathrm{His}1}^{\delta }$ bonds and A_33_ is the axial component (cf. Fig. 1).

**Table 3:** Effect of employed basis on the isotropic part (A_iso_) of HFCs (in MHz) for the coordinating N atoms with B3LYP (model 1 in Fig. 3, optimized without substrate and TPSS-D3/def2-SV(P) and MM region relaxed). CGTOs is the number of contracted Gaussian functions in the basis set.

Basis	# CGTOs	A_iso_ (${\text{N}}_{\mathrm{His}1}^{\delta }$)	A_iso_ (N_His1_)	A_iso_ (${\text{N}}_{\mathrm{His}78}^{∊}$)
				
aT/aT/aT/aT/aT	1910	36.1	39.1	36.3

B1: aTJ/T/T/T/D	1016	35.8	39.2	36.1
B2: aTJ/aTJ/T/T/D	1112	38.6	42.8	38.9
B3: aTJ/aTJ/aTJ/T/D	1240	38.6	42.8	38.9
B4: aTJ/aTJ/aTJ/aTJ/D	1416	38.6	42.8	38.9
B5: aTJ/aTJ/aTJ/aTJ/T	1677	38.6	42.8	38.9
B6: aTJ/aTJ/aTJ/aTJ/aTJ	1851	38.6	42.8	38.9

TZVP-uncontracted	1474	37.8	41.9	38.1
TZVPP-uncontracted	1725	37.5	41.6	37.8

Starting with the copper atom, Table 2 showed the expected large effect of using core-property basis sets on both the principal components, and (particularly) on the isotropic Fermi contact (A_FC_) term. The A_FC_ term changed by as much as 450 MHz and therefore also changed sign. The changes in some of the components of the hyperfine tensor, e.g. A_33_, were with about 1100 MHz even larger. Interestingly, the second-order spin-orbit contribution (${\text{A}}_{\mathrm{iso}}^{\mathrm{SO}}$) almost did not change: With the standard aug-cc-pVTZ basis set on all atoms it was 138 MHz, with the core-property basis set aug-cc-pVTZ-J on all atoms (B6) it was 136 MHz, and with the decontracted standard def2-TZVP and def2-TZVPP basis sets around 133 MHz. Slightly larger changes were observed on going to the smaller standard basis sets like cc-pVTZ and cc-pVDZ as shown in Table S2 in the SI. Yet, reasonable results (between 141 and 138 MHz) were still obtained. This basis set insensitivity of the spin-orbit contribution implies that the change with respect the employed basis set for the total isotropic hyperfine coupling constant (A_iso_) originates almost exclusively from the Fermi contact term. It is worthwhile to mention, that spin-orbit effects were (as expected) found to be large for the HFCs of Cu in LPMO: as one can see from Table 2, the second-order spin-orbit contribution (${\text{A}}_{\mathrm{iso}}^{\mathrm{SO}}$) is approximately 50% of the A_FC_ contribution but with opposite sign and thus essential to include in the calculations.

While it is clear that specialized basis sets must be employed on the Cu atom, employing aug-cc-pVTZ-J only on Cu gave values within 1 MHz of the results where all atoms had aug-cc-pVTZ-J basis sets. Additionally employing aug-cc-pVTZ-J on the first coordination sphere and the imidazole rings also had minimal effect. This shows that we can safely employ a local-dense strategy, thereby reducing the number of contracted functions considerably. In the following we will therefore continue with basis set B3. The result with this basis set, −116 MHz for $A_{\mathrm{iso}}$, differed by about 4 MHz from the results obtained with the decontracted standard triple zeta basis sets, def2-TZVP and def2-TZVPP, which were employed in Ref. [41], although this covers over differences of 7 MHz in the A_FC_ term and 3 MHz in the $A_{\mathrm{iso}}^{\mathrm{SO}}$ term with opposite signs.

Turning to nitrogen super HFCs, we see from Table 3 that as soon as one uses a core-property basis set on the nitrogen atoms of interest (B2), the choice of basis set on the other atoms does not make a difference anymore. The totally decontracted standard polarized triple zeta basis sets, def2-TZVP and def2-TZVPP, led to values for the super HFCs, which are 1 MHz smaller still.

We have also investigated various locally-dense combinations of the regular Dunning basis sets (shown in the SI, Tables cf. Tables S2 and S3), but the results were (as expected) erratic for the copper HFCs leading to errors of over 300 MHz compared to results with the aug-cc-pVTZ-J basis set on Cu, even when using the aug-cc-pVTZ basis set on all atoms (aT/aT/aT/aT/aT in Table 2). These results will therefore not be discussed in detail, although we note that reasonable results were obtained for HFCs of the nitrogen atoms with the regular Dunning basis sets.

### 3.3 The effect of functional and underlying structure

With the results from previous section in mind, we selected the basis set denoted “B3”, and proceeded to consider the effect of the employed DFT functional as well as the underlying structure on the calculated HFCs. For the latter, we first considered an indirect structural effect by employing QM/MM optimized structures with fixed MM region and compare these results to structures with (parts of) the MM region free; we further extended the size of the systems employed for the calculations of HFCs as shown in Fig. 3. These different structures were investigated with a range of DFT functionals to see how well the calculated HFCs (for a given structure and functional) reproduce the experimentally observed trend upon substrate binding. We also comment on how well the (absolute) experimental HFCs are reproduced by the various computational setups. In relation to comparison with experiment, it should be noted that we here investigated all (principal) components of the copper HFCs, although the fitting used to extract the HFCs from experimental spectra usually only allows accurate determination of $\left|A_{z}\right|$ (here denoted A_33_). Regarding the nitrogen HFCs, only the isotropic values could be resolved for the investigated *Ls*(AA9)A enzyme, and no assignment to individual nitrogen atoms was achieved [40].

#### 3.3.1 Hyperfine coupling constants from structures with fixed MM region

**Fig. 4:**
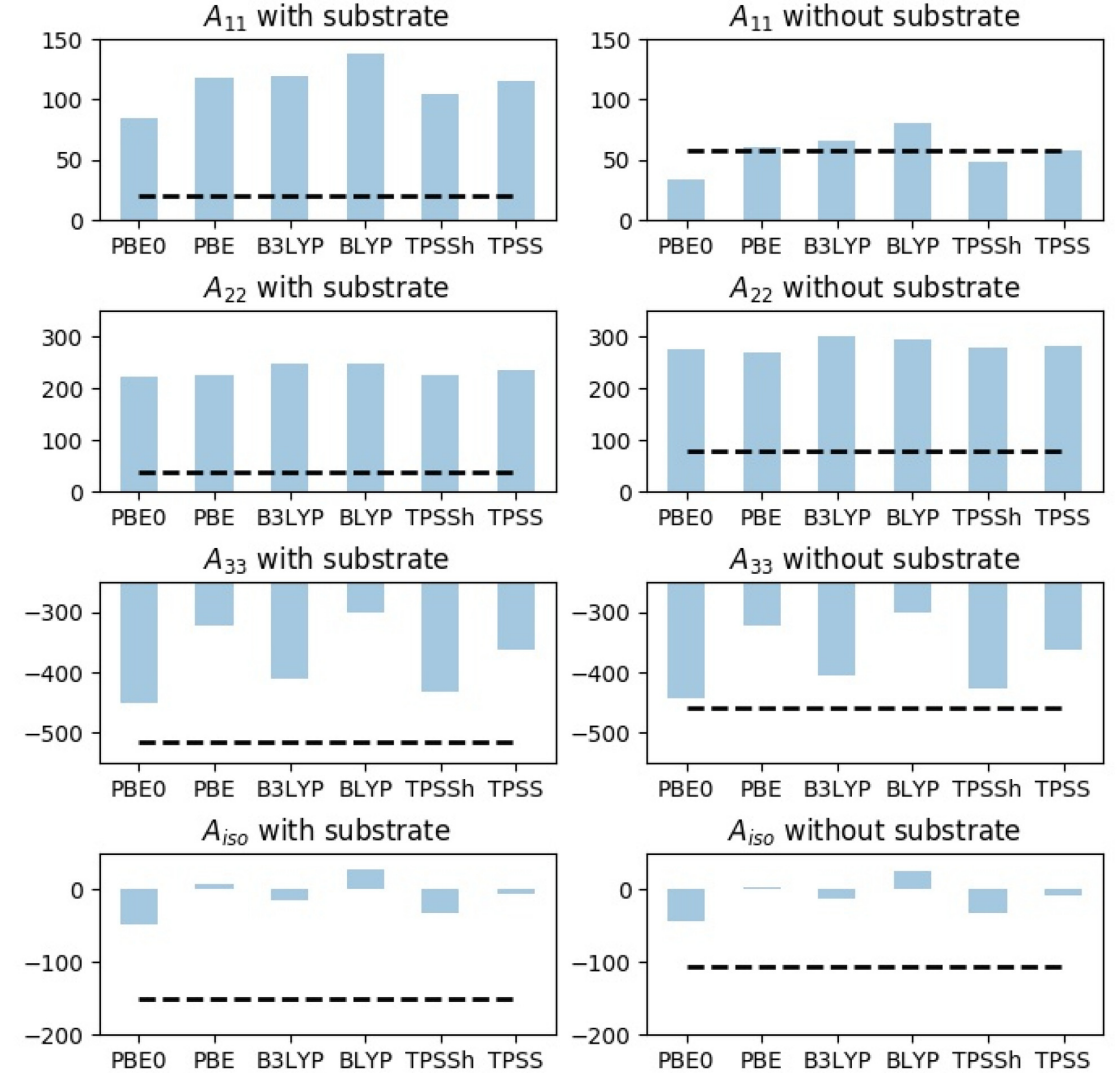
Calculated HFCs (in MHz) for Cu over six different functionals (for the individual principal components, A_11_–A_33_), all calculated on model 1 (see Fig. 3) with underlying structure QM/MM structures optimized with TPSS-D3/def2-SV(P) and the MM region fixed. Black dashed lines are experimental values (taken from Ref. [40]).

We first investigated the copper HFCs for substrate-free and substrate-bound states, respectively, when employing a structurally fixed MM region (and model 1 in Fig. 3). The HFCs for copper with this setup are shown in Fig. 4 and the underlying values are provided in Table 4. The calculated copper HFCs were found to deviate significantly from the experimental values for all three components of the HFC tensor for structures with substrate present (see Fig. 4); the $A_{11}$ and $A_{22}$ components were severely overestimated, while the $A_{33}$ component was severely underestimated (in absolute values). While the results were better for $A_{11}$ for the substrate-free structure, both $A_{22}$ and $A_{33}$ still show large deviations (with most functionals) from the experimental values.

**Table 4:** The three principal components (A_11_–A_33_)${}^{a)}$ of the Cu HFCs (in MHz) calculated with different functionals and basis set B3 (see Computational Details). The contributions from the Fermi contact (A_FC_) and spin–orbit (${\text{A}}_{\mathrm{iso}}^{\mathrm{SO}}$) terms are also given. All calculations were done on model 1 (see Fig. 3), obtained with an underlying structure from QM/MM TPSS-D3/def2-SV(P) with fixed MM region.

**With substrate**	A_FC_	${\text{A}}_{\mathrm{iso}}^{\mathrm{SO}}$	A_11_	A_22_	A_33_	A_iso_
PBE0	−198.4	150.0	84.6	221.7	−451.4	−48.4
PBE	−89.5	97.2	118.3	225.9	−321.3	7.7
B3LYP	−151.6	136.7	119.8	247.1	−411.4	−14.9
BLYP	−66.4	95.0	138.1	248.3	−300.5	28.6
TPSSh	−140.0	106.5	104.4	226.3	−431.1	−33.5
TPSS	−95.1	90.1	114.6	232.9	−362.7	−5.0

Exp. [40]	–	–	20	38	−515	−152

**Without substrate**	A_FC_	${\text{A}}_{\mathrm{iso}}^{\mathrm{SO}}$	A_11_	A_22_	A_33_	A_iso_

PBE0	−198.1	153.4	33.1	275.1	−442.4	−44.7
PBE	−96.6	98.7	60.3	269.0	−323.3	2.0
B3LYP	−153.0	139.9	65.4	300.1	−404.6	−13.0
BLYP	−72.2	96.6	80.7	293.6	−301.1	24.4
TPSSh	−142.0	108.7	47.9	279.2	−427.2	−33.4
TPSS	−99.4	91.5	57.7	280.9	−362.2	−7.9

Exp. [40]	–	–	58	78	−458	−107

${}^{a)}$ A_11_ is aligned with the N_His1_$-\text{Cu}-{\text{O}}_{W}^{\mathrm{eq}.}$ bonds; A_22_ is aligned with the ${\text{N}}_{\mathrm{His}78}^{∊}-\text{Cu}-{\text{N}}_{\mathrm{His}1}^{\delta }$ bonds and A_33_ is the axial component (cf. Fig. 1).

From Table 4 (and Fig. 4) we further see that the change in HFCs observed when the substrate binds could not always be reproduced. For instance, the change of A_33_ values was estimated considerably too small and sometimes in the wrong direction: we obtained changes between 7 and -2 MHz, compared to the experimental value of 57 MHz. Regarding the other two components of the HFC tensor, the A_11_ values all increased (between 51 and 57 MHz, cf. Table 5), whereas experimentally it is the opposite: they decreased with 38 MHz [40]. The qualitative changes for A_22_ were reasonably well reproduced, with an decrease of 48–54 MHz, compared to the experimental 40 MHz. However, it is clear from Fig. 4 that this is due to error compensation since (as mentioned above) the A_22_ values were significantly overestimated in both substrate-bound and substrate-free structures.

**Table 5:** The three principal components (A_11_–A_33_)${}^{a)}$ of the Cu HFCs (in MHz) calculated with different functionals and basis set B3. The contributions from the Fermi contact (A_FC_) and spin–orbit (${\text{A}}_{\mathrm{iso}}^{\mathrm{SO}}$) terms are also given. All calculations are on QM/MM structures, optimized with TPSS-D3/def2-SV(P) where the MM region is allowed to relax. The calculations on the substrate-bound state were done with three models of different sizes (cf. Fig. 3).

**With substrate**	A_FC_	${\text{A}}_{\mathrm{iso}}^{\mathrm{SO}}$	A_11_	A_22_	A_33_	A_iso_
	Model 1					
PBE0	−323.7	142.5	6.2	42.9	−592.7	−181.2
PBE	−213.3	91.9	21.7	75.2	−461.1	−121.4
B3LYP	−282.4	129.7	30.9	68.0	−557.1	−152.7
BLYP	−192.6	90.0	39.0	96.2	−443.0	−102.6
TPSSh	−269.2	101.8	5.5	52.8	−570.4	−167.4
TPSS	−222.2	86.0	22.0	72.6	−503.3	−136.2

	Model 2					
PBE0	−333.1	140.8	−0.4	25.4	−602.0	−192.3
PBE	−223.5	91.0	19.6	57.2	−474.3	−132.5
B3LYP	−293.0	128.0	23.6	48.6	−567.3	−165.0
BLYP	−203.6	89.1	36.4	77.6	−457.5	−114.5
TPSSh	−279.1	100.6	9.9	34.0	−579.4	−178.5
TPSS	−232.9	85.1	19.2	53.2	−515.8	−147.8

	Model 3					
PBE0	−335.8	138.1	−8.0	18.4	−603.7	−197.8
PBE	−227.0	89.2	21.8	45.5	−480.7	−137.8
B3LYP	−296.0	125.2	15.8	40.8	−568.7	−170.7
BLYP	−207.4	87.3	36.7	67.2	−464.0	−120.1
TPSSh	−280.8	98.6	4.9	26.7	−578.1	−182.2
TPSS	−235.2	83.4	20.0	42.5	−517.9	−151.8
Exp. [40]	–	–	20	38	−515	−152

**Without substrate**	A_FC_	${\text{A}}_{\mathrm{iso}}^{\mathrm{SO}}$	A_11_	A_22_	A_33_	A_iso_

	Model $2^{\prime }$					
PBE0	−305.7	147.4	13.3	82.1	−570.3	−158.3
PBE	−204.7	97.3	49.7	95.2	−467.1	−107.4
B3LYP	−264.3	134.3	40.6	105.9	−536.4	−130.0
BLYP	−183.3	95.6	69.0	119.1	−451.1	−87.7
TPSSh	−252.7	105.1	27.7	85.2	−555.7	−147.6
TPSS	−210.9	89.7	46.2	94.4	−504.2	−121.2
Exp. [40]	–	–	58	78	−458	−107

${}^{a)}$ A_11_ is aligned with the N_His1_$-\text{Cu}-{\text{O}}_{W}^{\mathrm{eq}.}$ bonds; A_22_ is aligned with the ${\text{N}}_{\mathrm{His}78}^{∊}-\text{Cu}-{\text{N}}_{\mathrm{His}1}^{\delta }$ bonds and A_33_ is the axial component (cf. Fig. 1).

In next section, we discuss QM/MM structures with the MM region relaxed, but a preliminary assessment of the importance of the underlying structure can be done by comparing the B3LYP results in Table 4 with the B3 results from Table 2 (which also were done with model 1 and B3LYP, but based on a structure optimized with the MM part relaxed). From this, we see that the underlying structure to be pertinent and the structures based on QM/MM optimization with relaxed MM region seem to perform significantly better than the unrelaxed counterpart: in particular, an approximately axial A-tensor was obtained in Table 2, while all functionals in Table 4 led to an A-tensor with pronounced rhombicity ($A_{11}\;\neq \;A_{22}\;\neq \;A_{33}$), which does not commensurate with experiment [40]. We can also make a preliminary estimate of the importance of the chosen functional: as expected, the functionals in Table 4 yielded HFCs that are significantly different between different choices, but a more thorough discussion is postponed to next section, since none of the functionals obtained qualitatively correct results based on a fixed MM region.

Finally, we also comment on the nitrogen (super) HFCs. Here only isotropic values have been obtained for the LPMO targeted in this study [40]. The values obtained from the experimental spectrum with the substrate bound were 36, 30 and 19 MHz. Our calculated values (Table S4) were in the range 30–48 MHz and thus in reasonable agreement with experiment. Similarly, without substrate we obtained quite similar values (35, 41 and 36 MHz). In this case, it was only possible to experimentally resolve two of the nitrogen HFCs (37 and 34 MHz) [40]. We again postpone the discussion to the use of more accurate structures below, but we note that nitrogen HFCs were much less sensitive to the underlying structure than copper HFCs.

#### 3.3.2 Hyperfine coupling constants from structures with free MM region

**Fig. 5:**
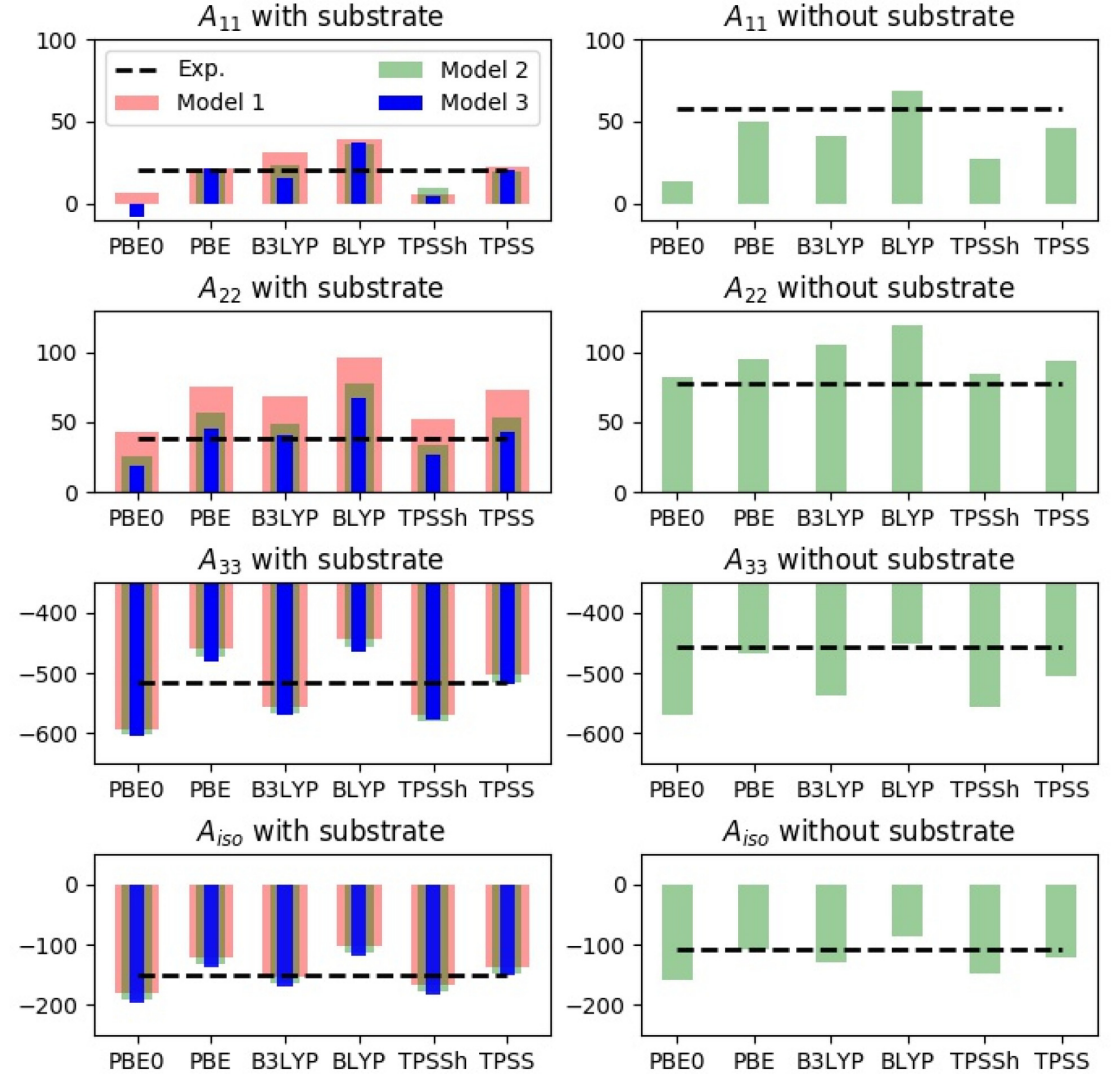
Calculated HFCs (in MHz) for copper over six different functionals (for the individual principal components, A_11_–A_33_), all calculated on QM/MM structures optimized with TPSS-D3/def2-SV(P) and the MM region allowed to relax. The calculations on the substrate-bound state were done with three models of different sizes (cf. Fig. 3). Experimental values are taken from Ref. [40].

Results based on structures obtained from QM/MM methods, where the MM region is allowed to structurally relax are shown in Fig. 5 and concrete values are given in Table 5. As implied in last section, we obtained for (the smallest) model 1 values that were overall in better agreement with experiment, compared to results with the MM region fixed (this holds for all functionals). To ensure that the better correspondence with experiment was not a fortuitous result due to the chosen system size, we included first the “pocket” water molecule [40] connecting the terminal NH_2_ group of His1 with the substrate through hydrogen bonding (model 2 in Fig. 3). Next, we included parts of the substrate (model 3) to investigate if the substrate had a direct effect on the electronic structure, and hence on the HFCs. The HFCs calculated with these three model sizes are shown in different colored bars in Fig. 5 (and are also provided in Table 5). Generally, the largest effect was seen when including the water molecule (i.e. between models 1 and 2), most pronounced for A_22_. Still, for all model sizes, the results were in general in better agreement with experimental values, compared to results for the fixed structure. Thus, the method employed in optimization of the underlying structure is among the most critical factors for accurate HFCs; the same holds true for the employed DFT functional, which we now will investigate in more detail. We focus here on the largest models i.e. model 3 for the substrate-bound structure and model 2 for the structure without substrate, cf. Fig. 3. For these models all functionals reproduced the changes in the A-tensor upon substrate binding observed experimentally, at least qualitatively. Thus, the absolute values of the A_33_ component was experimentally [40] found to increase with 57 MHz upon substrate binding, while we obtained calculated values between 14 and 32 MHz (also increasing). Meanwhile, for A_11_ the experimental value decreased 38 MHz upon substrate binding, while our theoretical values were a decrease between 21 and 33 MHz, depending on the functional (which is in reasonable correspondence with the experiment). Similarly, for A_22_ the calculated values decreased with 50–64 MHz, compared to the experimentally measured decrease of 40 MHz.

While the results generally reproduced the experimentally observed change in HFCs for the copper atom, we will still comment on the large differences in the absolute values between the functionals. For both substrate-bound and unbound states, individual differences between the functionals far surpassed differences induced by the chosen model size. Starting with the substrate-bound structure, the absolute value of A_33_ was the largest of the three HFC components and also where we obtained the largest differences between the different functionals, ranging from overestimation of 89 MHz (PBE0) to underestimation of 32 MHz (BLYP). For A_11_ all functionals, except PBE0, obtained results in reasonable agreement with the 20 MHz, obtained experimentally [40]. A general trend is that the inclusion of exact exchange into the functionals seem to lower the obtained value: for PBE0 this lowering led to a negative sign, and PBE0 is also in largest disagreement with experiment in absolute numbers (28 MHz). The results for A_22_ were also overall reasonable; the largest difference was here obtained for BLYP, overestimating the experimental results by 29 MHz. Again, we note that inclusion of exact exchange always led to increase of A_22_.

Moving to the unbound state, the different functionals showed somewhat larger scatter, suggesting this state is in fact more difficult than the bound state. The A_33_ value was the largest and also showed the largest spread in values: thus BLYP underestimated (in absolute numbers) the value with 7 MHz, while PBE0 overestimated with 112 MHz. Including exact exchange generally led to an increase in the absolute values. The A_11_ component spanned values from 13 MHz with PBE0 (underestimation of 44 MHz) to 69 MHz with BLYP (overestimation of 11 MHz). The values for A_22_ were generally lower than the experimental value, ranging from a value of 119 MHz (41 MHz from the experiment) with BLYP to 82 MHz with PBE0, only 4 MHz from experiment. Functionals including exact exchange always gave larger values, compared to the same functional without exact exchange.

As noted in a previous section, the nitrogen HFCs were much less dependent on the computational setup. Thus, values obtained with underlying QM/MM structures with the MM region relaxed (Table S5),were rather similar to the HFCs obtained from underlying QM/MM structures obtained with the MM region fixed. Moreover, the values in Tables S4 and S5 showed that nitrogen HFCs are fairly independent of the employed functional.

The nitrogen HFCs for the substrate-bound complex and the active site without substrate are similar in absolute size to the values obtained in Ref. [40]. However, without an experimental assignment to the individual atoms, it is difficult to make more precise comparisons. Intriguingly, our values in Table S5 are in quite good correspondence with the values obtained with DFT (PBE0) for a substrate-bound AA10 LPMO (*Sm*AA10A) in Ref. [41]: here they obtained 39 MHz for A_iso_ of N_His1_ and 32 MHz for A_iso_ of both $N_{\mathrm{His}1}^{\delta }$ and ${\text{N}}_{\mathrm{His}78}^{∊}$ (using the nomenclature for *Ls*(AA9)A). Notably, these values were used in fitting of experimental spectra, yielding simulated spectra that closely resembled the experimental ones. Hence, it would be interesting to employ the values obtained here in a similar fashion.

We have also calculated copper and nitrogen HFCs on model 3, employing an underlying structure obtained with def2-TZVPD (see Fig. 2). We will not discuss the results in the same detail, and we have compiled the resulting HFCs in the SI (Tables S6 and S7). We note that the structure of the substrate-bound state was close to unchanged and as expected, quite small changes in the obtained HFCs were therefore seen. However, the structure of the unbound state changed (as described in a previous section), mainly around the Cu–O bonds from the water molecule and tyrosine. As one might expect, this also affected the obtained HFCs for copper (again, the changes in nitrogen HFCs were much smaller). The HFCs were still qualitatively correct: for instance, the absolute value of A_33_ generally increased upon substrate binding (between 123 and 136 MHz), but were often in less good agreement with the experimental values, compared to calculations carried out on structures optimized with def2-SV(P). Particularly the A_22_ values are too large making the A-tensor more rhombic than axial; while it may seem that def2-SV(P) is the better choice, we should emphasize that to obtain results directly comparable with experiment, inclusion of system dynamics is pertinent, particular as the Cu–O bonds are rather weak and the calculated copper HFCs are highly dependent on these bonds.

In comparison to AA10 LPMOs, the decreasing A_11_ and A_22_ values, accompanied by an increasing A_33_-value upon substrate binding have also been observed for the *Sm*AA10A and *Bl*LPMO10A [41], [44]; DFT was able to qualitatively reproduce the observed changes, although large differences in absolute values were seen with respect to experiment. Our results here show that the findings from AA10 LPMOs can be transferred to *Ls*(AA9)A as DFT also in our case reproduced the observed trend upon substrate binding (although it was highly dependent on the used starting structure). In Ref. [44], the different contributions to the HFCs were analyzed and it was found that the main change was due to the A_FC_ term. Our results agreed that the changes in the A_FC_ term were the largest upon substrate binding (22–26 MHz), compared to the contributions from ${\text{A}}_{\mathrm{iso}}^{\mathrm{SO}}$ (7–9 MHz), as seen from Table 5. We also found the A_FC_ term to be more negative after substrate binding (the spin-orbit contribution from $A_{\mathrm{iso}}^{\mathrm{SO}}$ is positive and increases), as discussed in Ref. [44]; thus the changes of the HFCs seem to operate through the same mechanism in *Ls*(AA9)A and *Bl*LPMO10A.

In a broader perspective, we can compare our results to the experimental EPR results complied by Vu and Ngo [38], who divided LPMOs into three different zones in the Peisach-Blumberg plot, mainly due to different values of Cu(II) $\left|A_{z}\right|$: The AA10 family usually have trigonal bypyramidal coordinated Cu(II) and thus significantly lower values of $\left|A_{z}\right|$ (Zone 1, 330 ⩽ $\left|A_{z}\right|$ ⩽420 MHz). Most other LPMOs not bound to substrate or ligands have higher $\left|A_{z}\right|$ values (Zone 2, 420 ⩽ $\left|A_{z}\right|$ ⩽ 510 MHz), while binding to substrate further increases $\left|A_{z}\right|$ (Zone 3, above 510 MHz). Later work on AA14 [11] places it in Zone 2, while the AA15 EPR spectra were complex, with several species [12]. Focusing on calculations for the largest models in Table 5 (model 3 for substrate-bound and model 2′ for substrate-unbound structures), we see that the three hybrid functionals (PBE0, B3LYP, and TPSSh) generally led to calculated values for the unbound structure in Zone 3, while PBE and BLYP gave absolute values calculated for the substrate-bound LPMO below the Zone 3 range. Thus, TPSS is the only functional that consistently was within Zones 2 and 3 for the bound- and unbound states respectively. Calculations with fixed structures (Table 4) generally gave values outside these experimental ranges, underpinning the need for relaxing the MM regions during structure preparation.

## 4 Conclusion and outlook

We have calculated copper and nitrogen HFCs for the active site of the LPMO *Ls*(AA9)A, both in a substrate-unbound and a substrate bound state. The HFCs change upon substrate binding, and we have investigated whether DFT can reproduce the experimental trend. We find that this is possible, but the quality of the result is highly dependent on the computational setup. As expected, it is important to employ core-property basis sets in these calculations, otherwise the values for the Fermi contact and spin-dipolar contributions will be far off. However, these basis sets are only necessary for the atoms, which couplings are to be calculated, i.e. the use of locally-dense basis sets allows to reduce the size of the basis sets significantly. Perhaps more importantly, it is crucial to allow the MM region to relax (we use 6 Å from the QM region): employing a structurally relaxed MM region gives copper A-tensors that are approximately axial (for all functionals employed), while optimizations with the MM region fixed led to A-tensors where the principal components where very different (rhombic). In the latter case, some functionals also fail to reproduce the observed trend upon substrate binding. Nitrogen HFCs are much less sensitive to computational setup, and are generally obtained in reasonable correspondence to experimental values.

All employed functionals predicted qualitatively correct changes of the HFCs upon substrate binding (if structures with partly relaxed MM region are used). However, there are considerable differences between the individual functionals, and at present it seems best only to rely on DFT to reproduce such trends, rather than the absolute values. For the absolute values, we find that both PBE and TPSS perform well for the non-hybrid functionals while B3LYP and TPSSh perform well for the hybrid functional. Comparing with a broader selection of experimental EPR data, LPMOs have been divided into Zones 1–3, depending on where the $\left|A_{z}\right|$-values [38] falls within the Peisach-Blumberg plot. The TPSS functional is the only functional led to a correct categorization with $\left|A_{z}\right|$-values in Zone 2 for the unbound state while changing to Zone 3 for the bound state (as seen experimentally). However, several limitations posed by theoretical model may change the ordering of functionals and before addressing which of the functionals perform best, it would be desirable to address these limitations. For instance, system dynamics should be included since large structural differences were obtained for the weak Cu–O bonds, which again have large influence on the obtained HFCs. Alternatively, the functional performance could be probed against high-accurate theoretical data (e.g. two or four component relativistic methods). Regarding the latter, recent results for Cu(II) HFCs in 20 complexes showed that MP2 and CCSD calculations on average do not really perform better than some of the DFT functionals [110]. Further measures to improve the accuracy (within a one-component DFT framework) would be to employ a scalar-relativistic Hamiltonian, e.g., the zeroth order regular approximation (ZORA). Finally, our present calculations have not considered the direct electrostatic effect of the protein. Results from blue copper proteins [47] indicate that relativistic effects at the ZORA level as well as electrostatic effects are non-negligible (both between 10 and 30 MHz), but generally below the differences we find between the different DFT functionals.

## CRediT authorship contribution statement

**Yusuf A. Theibich:** Data curation, Investigation. **Stephan P.A. Sauer:** Conceptualization, Data curation, Investigation, Supervision, Writing - review & editing. **Leila Lo Leggio:** Conceptualization, Supervision, Writing - review & editing. **Erik D. Hedegård:** Conceptualization, Methodology, Writing - original draft, Visualization, Formal analysis, Writing - review & editing.

## Declaration of Competing Interest

The authors declare that they have no known competing financial interests or personal relationships that could have appeared to influence the work reported in this paper.
