# Supplementary material for: Estimating the accuracy of calculated electron paramagnetic resonance hyperfine couplings for a lytic polysaccharide monooxygenase
Source: Comput Struct Biotechnol J. 2020 Dec 20;19:555–67. doi: 10.1016/j.csbj.2020.12.014 (PMC7807142; doi:10.1016/j.csbj.2020.12.014)
Supplement: Supplementary file 1 [file mmc1.pdf]

Supporting Information: Estimating the accuracy of calculated electron paramagnetic resonance hyperfine couplings for a lytic polysaccharide monooxygenase

Yusuf A. Theibich,† Stephan P. A. Sauer,† Leila Lo Leggio,† and Erik D. Hedegård*,‡

†*Department of Chemistry, University of University, Copenhagen*

‡*Division of Theoretical Chemistry, Lund University, Chemical Centre, P. O. Box 124, SE-221 00 Lund*

E-mail: erik.hedegard@teokem.lu.se

Table S1: Selected bond angles (°) for the active site of *Ls*(AA9)A. For brevity, we denote TPSS-D3 and B3LYP-D3 as TPSS and B3LYP, respectively, while we denoted def2-SV(P) and def2-TZVPD as SV(P) and TZVPD.

QM//MM model	$N^{\delta}_{His1}-Cu-N_{His1}$	$N_{His1}-Cu-N^{\epsilon}_{His78}$	$N^{\epsilon}_{His78}-Cu-O^{W}_{Eq.}$	$O^{W}_{Eq.}-Cu-N^{\delta}_{His1}$
	Optimized without substrate *Ls*(AA9)A.			
TPSS/SV(P)//Fixed	93.4	88.9	94.1	94.6
TPSS/SV(P)//Free	92.9	89.4	84.7	92.5
TPSS/TZVPD//Free	89.4	91.9	83.7	95.2
Exp. 5ACG[S1]	91.9	90.6	86.4	91.6
	Optimized with substrate *Ls*(AA9)A.			
TPSS/SV(P)//Fixed[S2]	92.8	92.2	84.5	88.5
TPSS/SV(P)//Free[S2]	94.0	88.3	94.9	82.8
TPSS/TZVPD//Free	94.1	88.8	94.2	82.6
Exp. 5ACF[S1]	90.3	90.2	-	-

Table S2: Basis set study on LPMO active site model 1, optimized with QM/MM TPSS-D3/def2-SV(P) and MM region relaxed. The atoms labelled N are in first coordination sphere, O_{Tyr} is the coordinating O from Tyr164, C_{Im}/N_{Im} are from the imidazole rings (from His1 and His78, respectively) while C and H are remaining Carbon and Hydrogen atoms.

Cu/{N&O_{Tyr}}/{C_{Im}&N_{Im}}/C/H	# CGTOs	A^{FC}	A^{SO}_{iso}	A_{11}	A_{22}	A_{33}	A_{iso}
D/D/D/D/D	538	95.1	141.8	-188.9	412.3	487.4	236.9
T/T/T/D/D	787	78.0	137.8	-212.0	387.9	471.3	215.7
T/T/T/T/D	963	78.0	137.8	-211.9	388.0	471.3	215.8
T/T/T/T/T	1224	77.5	137.8	-212.3	387.5	470.7	215.3
aT/aT/T/T/D	1084	73.5	138.0	-215.0	384.1	465.2	211.4
aT/aT/aT/T/D	1212	127.0	138.1	-161.1	437.8	518.5	265.1
aT/aT/aT/aT/D	1388	153.6	138.1	-134.4	464.4	545.2	291.7
aT/aT/aT/aT/T	1649	160.2	138.1	-127.8	471.1	551.6	298.3
aT/aT/aT/aT/aT	1910	198.2	138.3	-89.6	509.2	589.7	336.5
Exp.	-	-	-	58	78	-458	-107

Table S3: Basis set study on LPMO active site model 1, optimized with QM/MM TPSS-D3/def2-SV(P) and MM region relaxed. The atoms labelled N are in first coordination sphere, O_{Tyr} is the coordinating O from Tyr164, C_{Im}/N_{Im} are from the imidazole rings (from His1 and His78, respectively) while C and H are remaining Carbon and Hydrogen atoms.

Cu/{N&O_{Tyr}}/{C_{Im}&N_{Im}}/C/H	# CGTOs	A_{iso} (N^{δ}_{His78})	A_{iso} (N_{His78})	A_{iso} (N^{ϵ}_{His1})
D/D/D/D/D	538	35.2	40.1	35.6
T/T/T/D/D	787	35.8	39.2	36.1
T/T/T/T/D	963	35.8	39.2	36.1
T/T/T/T/T	1224	35.8	39.0	36.1
aT/aT/T/T/D	1084	35.7	39.1	36.0
aT/aT/aT/T/D	1212	36.0	39.1	36.3
aT/aT/aT/aT/D	1388	36.0	39.1	36.3
aT/aT/aT/aT/T	1649	36.1	39.0	36.3
aT/aT/aT/aT/aT	1910	36.1	39.1	36.3
Exp.		- 34	-	37

Table S4: Isotropic HFCs for the coordinating N atoms, obtained with B3 and employing six different functionals. All values are calculated from QM/MM structures optimized with TPSS-D3/def2-SV(P) and the MM region fixed (all on model 1).

**With substrate**	A_{iso} (N^{δ}_{His1})	A_{iso} (N_{His1})	A_{iso} (N^{ϵ}_{His78})
PBE0	34.5	42.8	30.4
PBE	34.3	46.5	32.4
B3LYP	36.0	46.0	31.8
BLYP	34.4	47.5	32.5
TPSSh	34.8	45.4	30.7
TPSS	33.5	45.6	31.0
**Without substrate**	A_{iso} (N^{ϵ}_{His1})	A_{iso} (N_{His1})	A_{iso} (N^{δ}_{His78})
PBE0	35.3	38.0	35.5
PBE	36.4	41.7	38.2
B3LYP	37.1	40.9	37.3
BLYP	36.7	42.5	38.5
TPSSh	36.0	40.6	36.2
TPSS	35.4	40.8	36.6

Table S5: Isotropic HFCs for the coordinating N atoms, obtained with B3 and employing six different functionals. All values are calculated from QM/MM structures optimized with TPSS-D3/def2-SV(P) and the MM region free.

**With substrate**	A_{iso} (N^{δ}_{His1})	A_{iso} (N_{His1})	A_{iso} (N^{ϵ}_{His78})
		Model 1	
PBE0	41.0	42.6	35.2
PBE	43.6	47.1	37.0
B3LYP	43.2	45.6	36.8
BLYP	44.1	48.2	37.2
TPSSh	41.8	44.8	35.8
TPSS	42.1	45.9	35.8
		Model 2	
PBE0	40.8	42.2	35.8
PBE	43.6	45.7	38.1
B3LYP	42.8	45.0	37.5
BLYP	44.1	47.0	38.4
TPSSh	41.5	44.0	36.5
TPSS	42.0	44.8	36.8
		Model 3	
PBE0	42.5	42.6	34.9
PBE	46.1	45.7	37.3
B3LYP	44.7	45.4	36.4
BLYP	46.7	47.1	37.5
TPSSh	43.1	44.2	35.4
TPSS	43.9	44.8	35.7
**Without substrate**	A_{iso} (N^{δ}_{His1})	A_{iso} (N^{Ter}_{His1})	A_{iso} (N^{ϵ}_{His78})
		Model 2	
PBE0	36.6	40.3	37.5
PBE	40.4	45.7	41.2
B3LYP	38.3	43.1	39.4
BLYP	41.0	47.2	42.0
TPSSh	37.4	42.3	38.0
TPSS	38.4	44.0	38.9

Table S6: Cu HFCs calculated with different functionals and basis set B3. The underlying structures are model 3, obtained with and underlying structure from QM/MM TPSS-D3/def2-TZVPD with relaxed MM region.

**With substrate**	A^{FC}	A^{SO}_{iso}	A_{11}	A_{22}	A_{33}	A_{iso}
PBE0	-334.9	133.6	-15.0	22.5	-611.4	-201.3
PBE	-227.0	87.0	19.9	50.0	-489.8	-140.0
B3LYP	-294.5	121.5	10.6	46.1	-575.6	-173.0
BLYP	-207.1	85.4	36.1	71.8	-473.1	-121.7
TPSSh	-280.3	96.0	-0.3	31.9	-584.4	-184.3
TPSS	-235.2	81.4	15.9	48.0	-525.1	-153.8
Exp.	-	-	20	38	-515	-152
**Without substrate**	A^{FC}	A^{SO}_{iso}	A_{11}	A_{22}	A_{33}	A_{iso}
PBE0	-227.5	149.6	31.6	210.6	-475.8	-77.9
PBE	-119.4	97.8	74.2	217.3	-356.0	-21.5
B3LYP	-183.4	136.2	64.6	232.9	-439.2	-47.3
BLYP	-95.44	95.6	94.6	240.4	-334.6	0.12
TPSSh	-172.0	106.3	51.4	211.8	-460.5	-65.7
TPSS	-126.0	90.2	68.6	221.2	-397.1	-35.8
Exp.	-	-	58	78	-458	-107

Table S7: Isotropic HFCs for the coordinating N atoms, obtained with B3 and employing six different functionals. All values are calculated from model 3 (model 2 without substrate), obtained from QM/MM structures optimized with TPSS-D3/def2-TZVPD and the MM region relaxed.

**With substrate**	A_{iso} (N^{ϵ}_{His1})	A_{iso} (N_{His1})	A_{iso} (N^{δ}_{His78})
PBE0	43.5	42.7	35.5
PBE	47.0	45.9	38.0
B3LYP	45.6	45.3	36.
BLYP	47.7	47.3	38.3
TPSSh	44.1	44.2	35.9
TPSS	44.9	44.8	36.4
**Without substrate**	A_{iso} (N^{ϵ}_{His1})	A_{iso} (N_{His1})	A_{iso} (N^{δ}_{His78})
PBE0	34.5	42.8	30.4
PBE	34.3	46.5	32.3
B3LYP	36.0	46.0	31.8
BLYP	34.4	47.5	32.5
TPSSh	34.8	45.4	30.7
TPSS	33.5	45.6	31.0

References

(S1) Frandsen, K. E. H.; Simmons, T. J.; Dupree, P.; Poulsen, J.-C. N.; Hemsworth, G. R.; Ciano, L.; Johnston, E. M.; Tovborg, M.; Johansen, K. S.; von Freiesleben, P.; Marmuse, L.; Fort, S.; Cottaz, S.; Driguez, H.; Henrissat, B.; Lenfant, N.; Tuna, F.; Baldansuren, A.; Davies, G. J.; Lo Leggio, L.; Walton, P. H. The molecular basis of polysaccharide cleavage by lytic polysaccharide monooxygenases. *Nat. Chem. Biol.* **2016**, *12*, 298–305.

(S2) Hedegård, E. D.; Ryde, U. Molecular mechanism of lytic polysaccharide monooxygenases. *Chem. Sci.* **2018**, *9*, 3866–3880.
